# The impact of childhood psychological maltreatment on self-referential and mother-referential processing: evidence from perception and memory

**DOI:** 10.3389/fpsyt.2025.1700814

**Published:** 2025-11-24

**Authors:** Jixian Wang, Lin Song, Minghui Liu

**Affiliations:** Department of Psychology, School of Education Science, Harbin Normal University, Harbin, China

**Keywords:** psychological maltreatment, self-referential effect, mother-referential effect, self-perceptual matching task, remember/know task

## Abstract

**Background:**

Childhood psychological maltreatment is considered one of the most severe risk factors for developing psychopathological issues in adulthood. Previous studies have preliminarily indicated that psychological maltreatment disrupts the quality of mother–child relationships, but it remains unclear whether this negative impact extends to cognitive processes.

**Methods:**

This study examined the impact of childhood psychological maltreatment on the subjective emotional connection and objective cognitive differentiation between self and mother. The Child Psychological Maltreatment Scale was used to select 35 participants in the maltreatment group and 35 controls. The Inclusion of Other in the Self Scale was used to assess the subjective connection between self and mother. Two experiments were conducted to investigate the effects of psychological maltreatment on cognitive differentiation at the perceptual and memory levels. Experiment 1 used a self-perceptual matching task to assess the impact of maltreatment on the processing of different referential information. Experiment 2 employed the Remember/Know task to examine the influence of emotional valence and referential type on personality trait word recognition.

**Results:**

The CPMS negatively predicted IOS scores, indicating that psychological maltreatment reduced the subjective connection between self and mother. Perceptual and memory experimental results showed that, compared to the control group, the psychological maltreatment group did not exhibit impaired cognitive differentiation between self and mother. At the memory level, the self-referential and mother-referential effects were stronger in the maltreatment group than in the control group, but were not modulated by emotion. Emotional modulation appeared in the control group’s preference for negative emotions.

**Conclusions:**

While experiences of psychological maltreatment during childhood may subjectively reduce the emotional connection between self and mother, they may not impair cognitive differentiation between self- and mother-related information at the perceptual and memory levels, nor do they exhibit a negativity preference at the memory level. This study provides new evidence for understanding the mechanisms through which childhood psychological maltreatment influences self-cognitive functions in adulthood.

## 1 Introduction

Childhood maltreatment has long been recognized as one of the most significant risk factors contributing to maladaptive development and psychopathology in children, with its detrimental effects often persisting into adulthood (1–4). Among the various forms of maltreatment, psychological maltreatment—though less directly injurious to physical health than sexual or physical abuse—often co-occurs with them and has enduring effects. It is a particularly underestimated, pervasive, and covert form of maltreatment (5–7). The American Professional Society on the Abuse of Children defines psychological maltreatment as a pattern of neglectful or recurrent behaviors by caregivers that causes significant harm to a child’s cognitive, emotional, social, interpersonal, and physical health, or developmental well-being (8).

Childhood psychological maltreatment is closely linked to the development of negative self-cognition. A recent meta-analysis found a significant negative correlation between childhood trauma exposure and self-concept, with psychological maltreatment or emotional abuse, physical abuse, and sexual abuse all included in the analysis, and higher exposure predicting poorer self-concept development (9). Among these forms, sexual abuse had the most detrimental impact, indicating that psychological maltreatment does impair self-concepts, although its effect may be relatively limited. In contrast, the influence of psychological maltreatment on self-cognition appears to be more subjective in nature. Children tend to internalize the adverse emotional experiences arising from psychological maltreatment, gradually forming a negative self-concept or self-evaluation. For instance, individuals exposed to higher levels of psychological maltreatment exhibit lower self-esteem, which predicts greater emotional and behavioral issues (7). These findings suggest that experiences of psychological maltreatment may undermine the stability of self-concept or self-representation.

As an important attachment figure and source of emotional support in early individual development, the mother plays a crucial role in a child’s growth. However, it remains unclear whether experiences of childhood psychological maltreatment disrupt the emotional connection between the self and the mother. According to the self-expansion model, a central human motivation in development is to incorporate the identities of close others—such as partners or parents—into one’s self-concept, with the degree of overlap between the self and close-other representations increasing as relational closeness deepens (10, 11). Undoubtedly, as the central figure in a child’s socialization process, the mother is one of the key targets of self-expansion. Compared with non-maltreated children, those who experience psychological maltreatment during childhood are more likely to develop insecure attachment relationships with their mothers, which hinders them from perceiving the mother as a source of comfort and protection and leads to the formation of more negative maternal representations (12, 13). A recent study with Chinese samples has further shown that individuals who experienced more frequent childhood maltreatment reported poorer mother–child relationship quality, reflected in lower levels of perceived emotional closeness (14). These findings suggest that individuals with psychological maltreatment histories not only develop more negative maternal representations during the process of self-expansion toward the mother but also perceive themselves as more emotionally distant from her. Integrating evidence from research on self-concept and self-esteem (7, 15), we hypothesize that experiences of psychological maltreatment may lead to more negative self- and mother-representations and reduce the degree of overlap between the two. To examine the degree of overlap between self- and mother-representations, the present study employed the Inclusion of Other in the Self Scale (IOS) for assessment. The IOS, derived from the self-expansion model, is a single-item pictorial measure that evaluates the perceived closeness between two identity representations by assessing the degree of overlap between two circles symbolizing the self and the other (16–19).

Although experiences of psychological maltreatment during childhood may damage the quality of the mother–child relationship, it remains unclear whether this negative impact extends to objective cognitive processing. The self-expansion model further assumes that individuals process information related to close others from a perspective similar to their own, suggesting that the distinction between self-referential and other-referential effects is moderated by closeness (20). Specifically, individuals exhibit greater cognitive differentiation for information related to others with whom they have lower levels of closeness (21). The self-referential effect is consistently observed across multiple cognitive processing levels, including perception and memory, where people process self-relevant information more efficiently than information related to others (22, 23). At the fundamental perceptual level, self-referential encoding facilitates the rapid and automatic processing of stimuli, showing a greater advantage over mother-referential encoding (22, 24). Bai et al. (2) found that individuals with a history of psychological maltreatment retain a stable self-referential effect, implying that experiences of psychological maltreatment may not be sufficient to disrupt the stability of self-representations at the perceptual level. Based on this, we hypothesize that psychological maltreatment may not be enough to affect the cognitive differentiation between the self and the mother at the perceptual level.

However, it also remains unknown whether these adverse effects manifest at a deeper level of processing—specifically, in memory—and whether they are influenced by emotional regulation. Unlike the automatic processing at the perceptual level, deep self-referential encoding at the memory level activates the self-schema, causing individuals’ task performance to be influenced by prior self-relevant experiences or autobiographical memories (25, 26). Evidence from the Remember/Know (R/K) task indicates that self-referential encoding enhances memory performance for contextual details (R judgments) rather than familiarity (K judgments), and that mother-referential encoding shares a similar advantage (23, 27–29). Because self-referential processing at the memory level draws more heavily on interactions between the self and the mother, we hypothesize that experiences of psychological maltreatment may, on the basis of reducing the perceived connection between self and mother representations, further enhance their cognitive differentiation at the memory level. On the other hand, for individuals with experiences of psychological maltreatment, traumatic events are often accompanied by negative emotions. Therefore, the modulatory role of emotional valence constitutes an important factor to examine. Most of the existing evidence derives from studies on autobiographical memory. Some studies suggest that maltreated individuals exhibit enhanced memory for contextual details of negative traumatic experiences, whereas other studies have found that they tend to process such experiences in a more overgeneralized manner—an inconsistency that remains unresolved (30–32). In terms of emotional regulation at the memory level, we further hypothesize that the cognitive differentiation between self and mother in individuals with a history of psychological maltreatment will be amplified under negative emotional conditions.

In summary, the present study aimed to address three primary questions. (a) Does psychological maltreatment reduce the perceived subjective connection between self and mother representations? (b) At the fundamental and automatic perceptual level, does psychological maltreatment affect the degree of cognitive differentiation between the self and the mother? (c) At the memory level involving deeper processing, does psychological maltreatment influence cognitive differentiation between the self and the mother, and is it modulated by emotion? Considering cultural differences and to better adapt the study to Chinese participants, the Childhood Psychological Maltreatment Scale (CPMS), developed and revised for the Chinese population, was used as a screening tool to classify participants into the psychological maltreatment and control groups (33). To examine the first question, we employed the IOS to assess the subjective connection between self and mother representations in both groups and examined whether CPMS scores predicted IOS scores. To address the second and third questions, two experiments were conducted. Experiment 1 used the self-perceptual matching task (SPMT) to investigate the influence of psychological maltreatment on the processing of different referential information while controlling for stimulus familiarity. Experiment 2 adopted the R/K task to examine how psychological maltreatment affects recognition performance for personality trait words varying in emotional valence and referential type. We proposed the following hypotheses.

Hypothesis 1: CPMS scores would negatively predict IOS scores.

Hypothesis 2: At the perceptual level, the difference between self-referential and mother-referential effects would not differ between the psychological maltreatment group and the control group.

Hypothesis 3: At the memory level, the psychological maltreatment group would exhibit a negativity preference in both the self-referential effect and the mother-referential effect. Additionally, the difference between these two effects would be larger in the maltreatment group compared to the control group, and this difference would be further amplified under negative emotional conditions.

## 2 Materials and methods

### 2.1 Experiment 1

#### 2.1.1 Participants

This study was approved by the Ethics Committee of the institution. Based on the interaction effect size reported by Bai et al. (2) (η2 p = 0.084, *f* = 0.303), *a priori* power analysis was conducted using G*Power 3.1 (ANOVA: repeated measures, within–between interaction), with α = 0.05 and 1 - β = 0.99. The results indicated that at least 42 participants were needed for Experiment 1. In Experiment 1, 70 participants were recruited through CPMS screening, including 35 in the psychological maltreatment group and 35 in the control group. All participants were right-handed, had normal or corrected-to-normal vision, and reported no color blindness or color weakness. Participants received appropriate compensation for their participation.

#### 2.1.2 Stimulus and materials

The CPMS primarily assesses childhood psychological maltreatment experiences by having participants recall their interactions with caregivers before the age of 18, including five dimensions: intimidation, neglect, belittlement, interference, and indulgence, with a total of 23 items. The scale uses a 5-point scoring system (scores range from 0 to 4), where higher scores indicate more frequent experiences of psychological maltreatment. A survey was conducted with 900 university students in Heilongjiang Province, yielding 827 valid responses, and Cronbach’s α was 0.9. From participants whose mean score exceeded 1.5, 35 individuals were randomly selected to constitute the psychological maltreatment group (age = 18.89 ± 1.80, 28 females, 7 males). From those whose mean score was below 1.0, 35 individuals were randomly selected to form the control group (age = 19.34 ± 1.66, 23 females, 12 males) (2, 34). In addition, the IOS was employed to measure the degree of subjective connection between the representations of the self and the mother. The IOS consists of seven pairs of circles that vary in degree of overlap. Greater overlap between the “self” and “mother” circles (scores range from 1 to 7, with higher values indicating greater overlap) reflected a stronger self-expansion of the mother concept (35).

Considering the Chinese cultural context, the name of a renowned literary figure, Lu Xun, was chosen as the label for the celebrity condition (28). In the SPMT, simple geometric shapes—triangle, square, and circle—were used, with the combinations of shapes and labels balanced across participants (22). The fixation cross subtended a visual angle of 0.38° × 0.38°. Geometric figures subtended a visual angle of 3.5° × 3.5°, and name labels subtended a visual angle of 2° × 1°. The inner edge of each geometric figure or name label was positioned 1.2°from the center of the screen.

#### 2.1.3 Procedure

**Figure 1:**
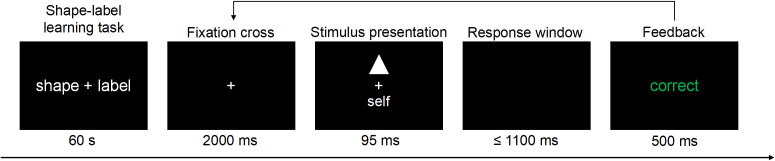
SPMT procedure flowchart for Experiment 1. The task consisted of two parts: a shape–label learning task and a matching judgment task. In the learning phase, participants were instructed to learn the associations between identity labels and geometric shapes during a 60s study period. The subsequent matching judgment phase proceeded as follows. Each trial began with a fixation cross presented for 2000 ms, followed by the simultaneous presentation of a figure and a label above and below the fixation cross. Participants were required to judge within 1100 ms whether the figure and label matched. Finally, feedback was presented for 500 ms.

The experiment was programmed using E-Prime 2.0, and stimuli were presented on a black screen with white visuals. The SPMT consisted of a learning phase, a practice phase, and a formal experimental phase. During the learning phase, participants were required to establish associations between different identity labels and geometric shapes, lasting for 1 minute. The practice phase included 18 trials, where each trial began with the presentation of a fixation point for 2000 ms, followed by the simultaneous display of a geometric shape and identity label above and below the fixation point for 95 ms. Participants were instructed to respond with a key press based on whether the label and shape matched. The response window was limited to a maximum of 1100 ms. After each trial, feedback (correct, incorrect, or too slow) was presented for 500 ms. The formal experimental phase was identical to the practice phase, consisting of 4 blocks, each containing 60 trials. After each block, accuracy feedback was presented, followed by a 15-second rest period. The procedure for Experiment 1 is shown in **Figure 1**.

#### 2.1.4 Data analysis

To examine Hypothesis 1, a simple linear regression model was constructed with the CPMS score as the independent variable and the IOS score as the dependent variable. To further examine Hypothesis 2, the celebrity reference condition was used as the baseline. The behavioral difference between the self-referential and celebrity-referential conditions was defined as the self-referential effect (SRE), whereas the difference between the mother-referential and celebrity-referential conditions was defined as the mother-referential effect (MRE) (18). According to previous studies using the SPMT, SRE occurs most consistently in matching trials (18, 22, 36, 37). Therefore, analyses typically focus on differences among referential conditions within the matching trials. For Experiment 1, reaction time (RT) and accuracy were each analyzed using a 2 (group: psychological maltreatment, control) × 2 (referential type: self, mother) repeated-measures ANOVA (rmANOVA). The presence of a significant interaction would indicate that the degree of cognitive differentiation between self and mother differs across groups.

### 2.2 Experiment 2

#### 2.2.1 Participants

Based on the interaction effect size reported by Bai et al. (2) (η2 p = 0.062, *f* = 0.257), *a priori* power analysis was conducted using G*Power 3.1 (ANOVA: repeated measures, within–between interaction), with α = 0.05 and 1 - β = 0.99. The results indicated that at least 36 participants were needed for Experiment 2. The participants in Experiment 2 were the same as those in Experiment. To control for potential interference effects between experiments, the two experiments were administered seven days apart, and the order of the experiments was counterbalanced across participants.

#### 2.2.2 Stimulus and materials

A total of 146 two-character personality trait adjectives were selected from the Modern Chinese Frequency Dictionary, including 74 positive adjectives and 72 negative adjectives. 22 undergraduate participants, who were not involved in subsequent experiments, rated their familiarity with these words using a 5-point scale (1 indicating unfamiliarity, 5 indicating very familiar). Additionally, participants rated the emotional valence of these words on a 7-point scale (1 indicating very negative, 7 indicating very positive). 60 positive adjectives and 60 negative adjectives were ultimately selected. There was no significant difference in familiarity between positive (4.75 ± 0.06) and negative adjectives (4.64 ± 0.09) (*p* > 0.05). However, there was a significant difference in emotional valence, with positive adjectives (6.18 ± 0.29) showing higher valence than negative adjectives (2.55 ± 0.54) (*p* < 0.01). 120 adjectives were randomly divided into two groups, each containing 60 adjectives, with an equal split between positive and negative words. One group was used as old items during the learning phase, while the other group served as new items during the recognition phase. A Latin square design was used to balance the reference conditions across the experimental conditions.

#### 2.2.3 Procedure

The R/K task was divided into three phases: the judgment task phase, the distraction task phase, and the recognition task phase. In the judgment task, a fixation point was presented for 1000 ms, followed by the presentation of the reference identities and adjectives. Participants were instructed to judge whether the presented personality trait word described a specific identity and respond by pressing a key: “1” for “yes, it describes” and “2” for “no, it does not describe,” with no time limit. A total of 60 adjectives were learned during this task. To prevent participants from rehearsing the items from the judgement task, a distraction task was introduced, requiring participants to complete simple mathematical calculation tasks for 3 minutes.

**Figure 2:**
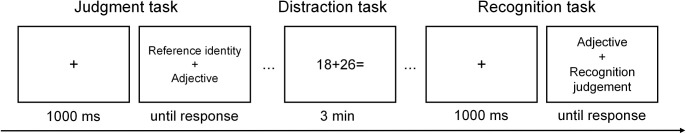
R/K task procedure flowchart for Experiment 2. The task comprised three phases: a judgment task, a distraction task, and a recognition task. In the judgment task, participants judged whether a given adjective could be used to describe a particular identity. In the subsequent distraction phase, participants solved a series of simple arithmetic problems, designed to prevent rehearsal of the studied items. The final recognition phase was an unexpected memory test, of which participants had not been informed beforehand. In this phase, they were asked to make R or K judgments for both previously studied and novel items.

It should be noted that the recognition task was an unexpected memory test, of which participants had not been informed beforehand. In the recognition task, adjectives were presented, and participants were asked to determine whether they had encountered the word during the judgement task. Participants pressed “1” for “yes, I have seen it” and “2” for “no, I have not seen it.” Afterward, participants made an R/K judgment for the words they recognized. In the R judgment, participants pressed “1” if they could consciously recall the details associated with the recognition process. In the K judgment, participants pressed “2” if they felt familiar with the word but could not recall the details. Finally, participants made a reference judgment for the words they classified as “R,” determining which identity the word had been used to describe in the judgment task. They pressed “1” for self, “2” for mother, and “3” for Lu Xun. The recognition phase included a total of 120 words, with 60 old words from the learning phase and 60 new words. All key press orders were counterbalanced across participants. The procedure for Experiment 2 is shown in **Figure 2**.

#### 2.2.4 Data analysis

To examine Hypotheses 3, SRE and MRE were also calculated. For Experiment 2, separate 2 (group: psychological maltreatment, control) × 2 (emotional valence: positive, negative) × 2 (referential type: self, mother) rmANOVA were conducted on the accuracy for R judgments and K judgments.

To further explore the relationships between subjective report scores and objective behavioral indicators, CPMS and IOS scores were treated as continuous variables, and their correlations with each behavioral measure were examined. In line with the study hypotheses, the difference between the SRE and MRE was used as an index of cognitive differentiation between self and mother. Subsequently, Pearson correlation analyses were conducted between subjective report scores and behavioral indicators.

## 3 Results

### 3.1 Descriptive statistics and regression analysis of CPMS and IOS

**Figure 3:**
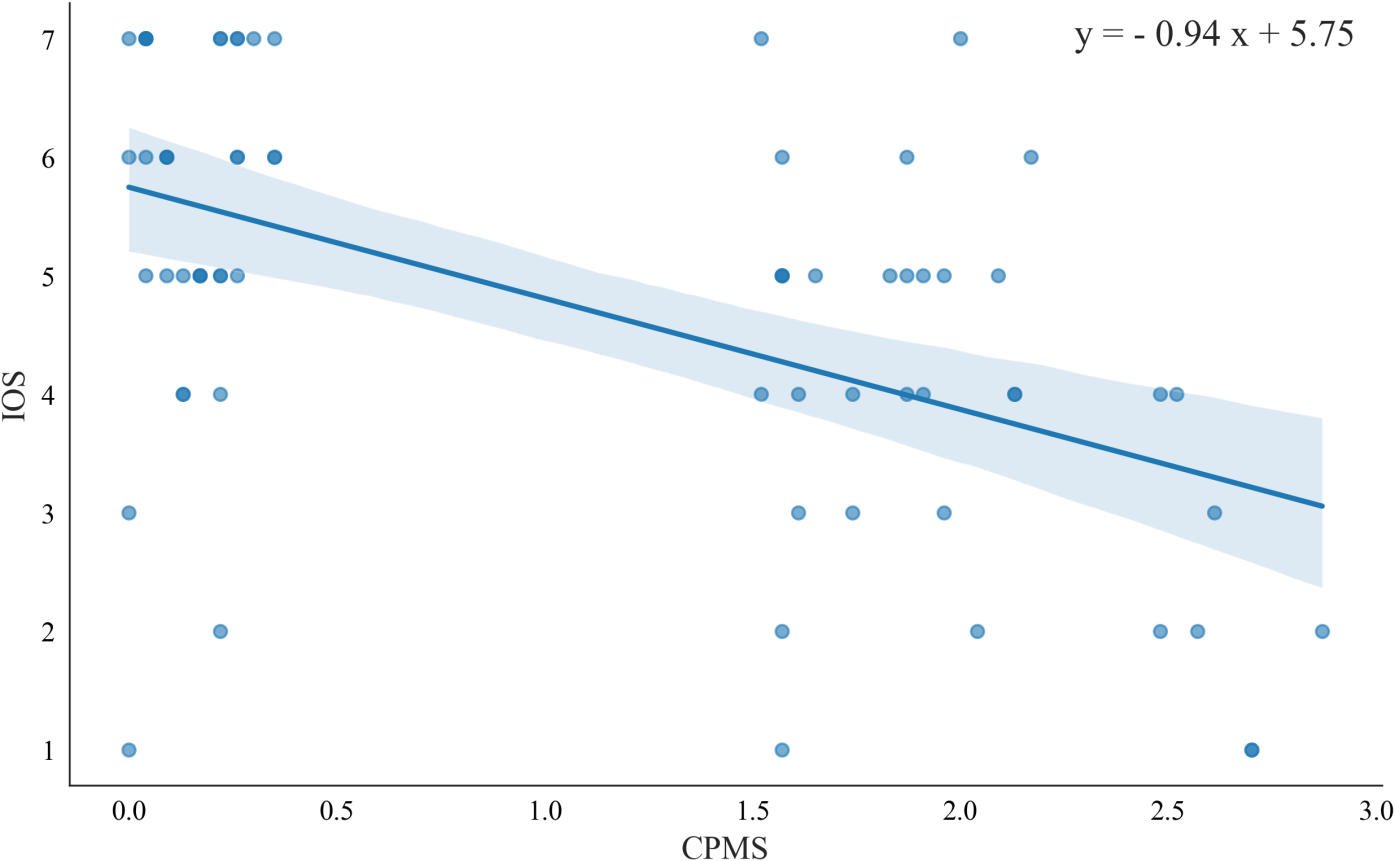
Linear regression fitting plot of IOS against CPMS. CPMS scores significantly and negatively predicted IOS scores, indicating that more frequent childhood psychological maltreatment experiences are associated with a lower degree of closeness between self and mother representations.

The results showed that CPMS scores significantly and negatively predicted IOS scores (β = −0.52, *t* = −5.01, *p* < 0.001), indicating that higher frequencies of psychological maltreatment during childhood were associated with a lower subjective connection between self and mother representations. Descriptive statistics and regression results for CPMS and its subdimensions in relation to IOS scores are presented in **Table 1**, and the linear regression fitting plot is shown in **Figure 3**.

**Table 1:** Descriptive statistics and regression results for IOS and CPMS.

Scale scores	CPMS	Intimidation	Neglect	Belittlement	Interference	Indulgence	IOS
Descriptive statistics results (M ± SD)							
Total scores	1.07 ± 0.97	1.28 ± 1.14	1.07 ± 1.11	0.94 ± 1.08	0.83 ± 1.03	1.06 ± 1.32	4.74 ± 1.74
Maltreatment group	1.98 ± 0.40	2.32 ± 0.62	2.01 ± 0.80	1.85 ± 0.80	1.52 ± 1.08	1.91 ± 1.38	3.94 ± 1.63
Control group	0.16 ± 0.11	0.25 ± 0.21	0.12 ± 0.15	0.04 ± 0.11	0.14 ± 0.17	0.21 ± 0.39	5.54 ± 1.48
Regression results							
Regression equation			Overall fitting index			Regression coefficient	
Dependent variable	Independent variable		*R*	*R^2^*	*F*	*β*	*t*
IOS	CPMS		0.52	0.27	25.13^***^	-0.52	-5.01^***^

*** p < 0.001.

To test group differences, independent-samples *t* tests were conducted to compare CPMS and IOS scores between the two groups. Results indicated that the psychological maltreatment group scored significantly higher on the CPMS than the control group, *t (68*) = 26.04, *p* < 0.001, Cohen’s *d* = 6.22. In contrast, the psychological maltreatment group scored significantly lower on the IOS, *t (68*) = −4.30, *p* < 0.001, Cohen’s *d* = −1.03.

### 3.2 Results of experiment 1

The results of RT revealed a significant main effect of referential type, *F*(1, 68) = 12.11, *p* = 0.001, η2 p = 0.15. The SRE (59.76 ± 8.18) was greater than the MRE (30.97 ± 8.03). The main effect of group (*F*(1, 68) = 0.11, *p* = 0.737, η2 p = 0.002) and the interaction between group and referential type [*F*(1, 68) = 0.01, *p* = 0.914, η2 p < 0.001] were both nonsignificant.

**Figure 4:**
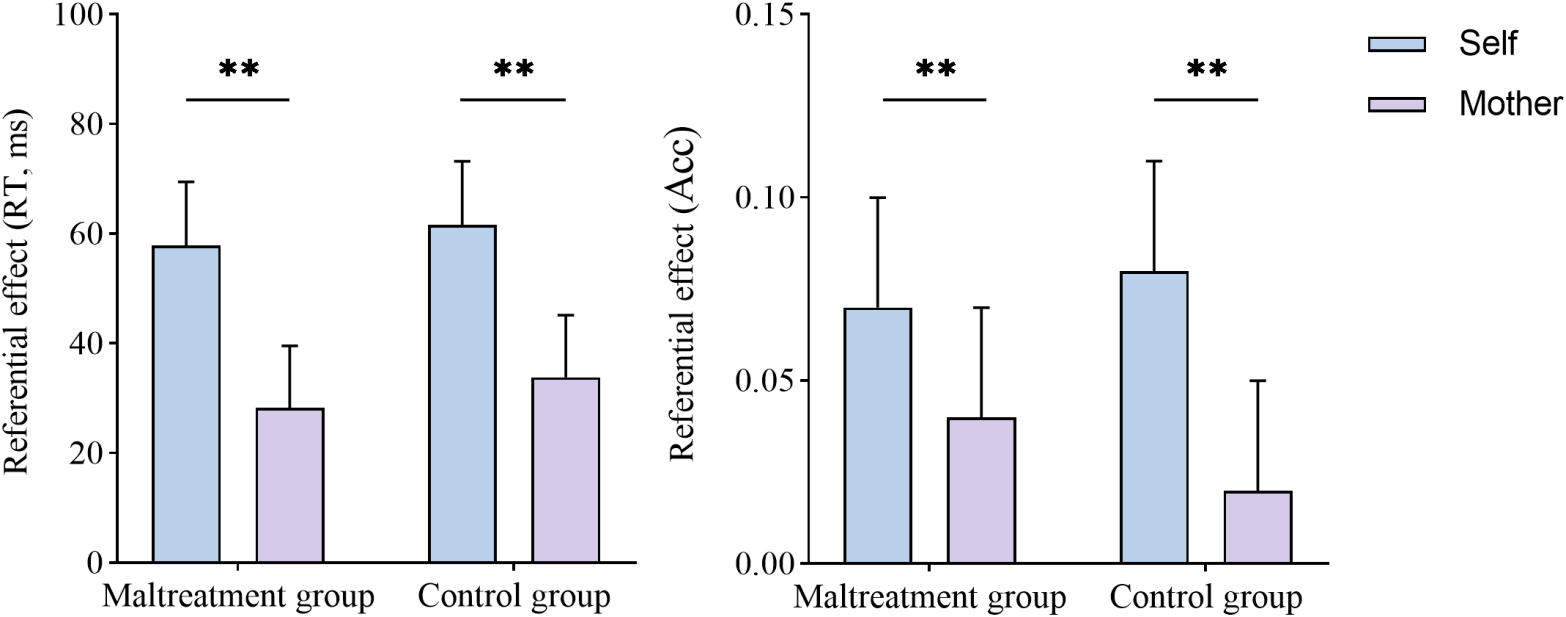
Referential effects in RT (left) and accuracy(right) in the matching trials of Experiment 1. The results of RT and accuracy showed that both the psychological maltreatment and control groups exhibited a stronger SRE than MRE. Error bars represent standard errors of the mean (SEMs). **p < 0.01. The same notation applies throughout.

The results of accuracy revealed a significant main effect of referential type, *F*(1, 68) = 8.65, *p* = 0.004, η2 p = 0.11. The SRE (0.08 ± 0.02) was greater than the MRE (0.03 ± 0.02). The main effect of group [*F*(1, 68) = 0.001, *p* = 0.974, η2 p < 0.001] and the interaction between group and referential type (*F*(1, 68) = 1.05, *p* = 0.310, η2 p = 0.02) were both nonsignificant. The results of Experiment 1 are shown in **Figure 4**.

### 3.3 Results of experiment 2

#### 3.3.1 R judgment accuracy

**Figure 5:**
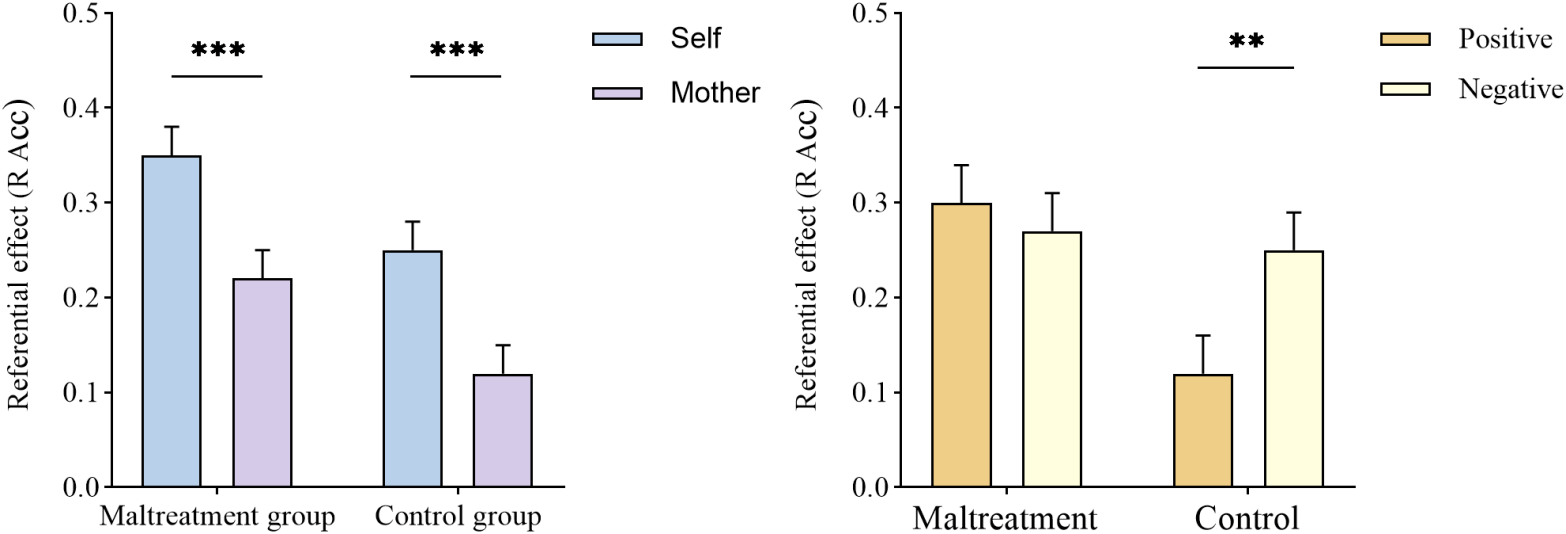
Referential effects in accuracy of R judgments in Experiment 2. The left panel depicts the interaction between group and referential type. Both the psychological maltreatment and control groups exhibited a stronger SRE than MRE. The right panel depicts the interaction between group and emotional valence. The control group showed a negativity preference in the referential effect, whereas the psychological maltreatment group was unaffected by emotional valence. ** p < 0.01, *** p < 0.001.

The results revealed a significant main effect of referential type, *F*(1, 68) = 37.63, *p* < 0.001, η2 p = 0.36. The SRE (0.30 ± 0.02) was greater than MRE (0.17 ± 0.02). The main effect of group was also significant, *F*(1, 68) = 5.85, *p* = 0.020, η2 p = 0.08. The psychological maltreatment group (0.29 ± 0.03) exhibited larger referential effects than the control group (0.19 ± 0.03). Regarding interactions, only the interaction between group and emotional valence was significant, *F*(1, 68) = 6.27, *p* = 0.015, η2 p = 0.08. The control group showed higher referential effects for negative words than for positive words (*p* = 0.005), whereas no emotional difference was observed in the psychological maltreatment group (*p* = 0.515). Besides, the main effect of emotional valence (*F* (1, 68) = 2.49, *p* = 0.119, η2 p = 0.04), the interaction between referential type and emotional valence (*F*(1, 68) = 0.11, *p* = 0.741, η2 p = 0.002), group and referential type (*F*(1, 68) = 0.001, *p* = 0.973, η2 p < 0.001) and the three-way interaction (*F*(1, 68) = 0.11, *p* = 0.741, η2 p = 0.002) were all nonsignificant. The R judgment accuracy results of Experiment 2 are shown in **Figure 5**.

#### 3.3.2 K judgment accuracy

The results revealed that all main effects and interaction effects were nonsignificant. The main effects of group (*F*(1, 68) = 1.46, *p* = 0.231, η2 p=0.02), referential type (*F*(1, 68) = 0.88, *p* = 0.352, η2 p = 0.01) and emotional valence (*F*(1, 68) = 2.65, *p* = 0.108, η2 p = 0.04) were all nonsignificant. The interaction between group and referential type (*F*(1, 68) = 0.22, *p* = 0.641, η2 p = 0.003), group and emotional valence (*F*(1, 68) = 3.39, *p* = 0.070, η2 p = 0.05), referential type and emotional valence (*F*(1, 68) < 0.001) and the three-way interaction (*F*(1, 68) = 0.47, *p* = 0.498, η2 p = 0.01) were all nonsignificant.

### 3.4 Correlation analysis between subjective report scores and behavioral indicators

The results revealed that CPMS scores were significantly associated with certain behavioral indicators under the R judgment condition in Experiment 2. Specifically, CPMS scores—as well as the dimensions of intimidation, belittlement, and indulgence—showed significant positive correlations with the SRE under the positive emotional condition. In addition, CPMS scores—as well as the dimensions of intimidation, neglect, belittlement, and indulgence—were significantly and positively correlated with the MRE under the positive emotional condition. These results suggest that individuals who experienced more frequent psychological maltreatment during childhood demonstrated stronger contextual detail memory for both self-referential and mother-referential information under positive emotional conditions. All other correlation coefficients were nonsignificant. The detailed results of the correlation analyses are presented in **Table 2**.

**Table 2:** Correlation results for scale scores and behavioral indicators.

Scale scores & behavioral indicators		CPMS	Intimidation	Neglect	Belittlement	Interference	Indulgence	IOS
Exp 1	SRE (RT)	-0.03	-0.08	-0.03	-0.05	0.06	0.03	0.10
	MRE (RT)	-0.07	-0.04	-0.11	-0.09	0.05	-0.12	0.15
	SRE - MRE (RT)	0.04	-0.03	0.08	0.05	0.01	0.14	-0.05
	SRE (ACC)	-0.13	-0.16	-0.08	-0.19	-0.05	-0.06	0.10
	MRE (ACC)	-0.03	-0.02	-0.01	-0.09	0.01	0.01	0.11
	SRE - MRE (ACC)	-0.14	-0.18	-0.10	-0.14	-0.07	-0.07	0.01
Exp 2	Positive SRE (Racc)	0.31^*^	0.35^**^	0.23	0.24^*^	0.10	0.38^**^	-0.23
	Positive MRE (Racc)	0.37^**^	0.38^**^	0.35^**^	0.31^**^	0.14	0.32^**^	-0.15
	Positive SRE - MRE (Racc)	-0.01	0.03	-0.08	-0.03	-0.02	0.11	-0.10
	Negative SRE (Racc)	0.03	-0.01	0.06	-0.03	0.04	0.09	0.02
	Negative MRE (Racc)	0.07	0.05	0.11	0.08	-0.04	0.10	0.21
	Negative SRE - MRE (Racc)	-0.04	-0.06	-0.04	-0.11	0.08	-0.01	-0.20
	Positive SRE (Kacc)	0.07	0.13	-0.04	0.09	0.05	0.03	-0.08
	Positive MRE (Kacc)	-0.05	0.01	-0.12	-0.04	-0.05	-0.02	-0.01
	Positive SRE - MRE (Kacc)	0.14	0.14	0.09	0.15	0.12	0.06	-0.08
	Negative SRE (Kacc)	-0.20	-0.23	-0.10	-0.20	-0.12	-0.19	0.13
	Negative MRE (Kacc)	-0.16	-0.18	-0.09	-0.16	-0.15	-0.08	0.11
	Negative SRE - MRE (Kacc)	-0.02	-0.03	-0.01	-0.03	0.05	-0.10	0.02

* p < 0.05, ** p < 0.01.

## 4 Discussion

The present study examined the impact of childhood psychological maltreatment on the subjective connection between self and mother representations and on the objective cognitive differentiation between them. The subjective report results indicated that psychological maltreatment experiences reduced, to some extent, the perceived connection between the self and the mother. The objective cognitive findings showed that psychological maltreatment did not increase cognitive differentiation between self-related and mother-related information at either the perceptual or the memory level. At the memory level, however, the psychological maltreatment group exhibited stronger SRE and MRE than the control group, and the modulation by emotional valence was manifested primarily as a negativity preference in the control group.

The subjective report results showed that CPMS scores significantly and negatively predicted IOS scores. This finding was consistent with Hypothesis 1, indicating that experiences of psychological maltreatment partially undermined the perceived emotional connection between self and mother representations. This result aligns with previous research and may be related to the insecure attachment patterns induced by early experiences of psychological maltreatment (12, 13). Building on traditional attachment theory, Riggs (38) proposed a lifespan attachment system model that incorporates psychological maltreatment. This model suggests that early psychological maltreatment leads to insecure attachment, which in turn impairs the quality of intimate relationships in adulthood. A growing body of evidence has demonstrated that childhood maltreatment contributes to the formation of insecure attachment patterns (12, 13, 39, 40). Individuals exposed to such experiences tend to struggle to perceive their mothers as stable sources of safety and protection, are more likely to develop negative mother-representations, and experience lower-quality mother–child relationships. From the perspective of the self-expansion model, the primary motivation for self-expansion is to maintain enduring and stable intimate relationships, which prompt individuals to incorporate information about close others as part of the self (10, 19). The present evidence suggests that psychological maltreatment may reduce closeness by disrupting secure attachment patterns, thereby weakening the emotional connection between the self and the mother (41).

The perceptual results supported Hypothesis 2, indicating that there is currently insufficient evidence to support that experiences of psychological maltreatment affect cognitive differentiation between self- and mother-related information. This suggests that the negative effects of psychological maltreatment may be confined to the subjective evaluation level, without extending to objective cognitive processing. From an evolutionary perspective, SRE offers a potential survival and developmental advantage, as it helps individuals prioritize processing information that is closely related to themselves, facilitating the approach of beneficial stimuli and avoidance of harmful ones (42). The perceptual results are consistent with Bai et al. (2), showing that individuals with psychological maltreatment history retain stable self- and mother-representations at the basic cognitive processing level. In the SPMT, participants were required to rapidly judge the identity information represented by geometric shapes. This process is primarily automated and requires minimal activation of existing experiences within the self-concept system. Therefore, negative self- and mother-representations related to maltreatment may not be activated early in information processing and do not appear to affect this basic cognitive function. It is important to note that the results of Experiment 1 cannot be attributed to familiarity effects. In the SPMT, the temporary link between social identity and geometric shapes maximized the exclusion of potential confounding effects from the familiarity associated with directly using identity stimuli.

Although no significant cognitive differentiation results were found at the memory level, we observed an enhancement of both SRE and MRE in the psychological maltreatment group. Previous research has found no difference in memory ability between maltreated children or adults with a history of childhood maltreatment and control groups (31, 43). This is largely consistent with Goodman et al. (30), suggesting that unless maltreatment is severe enough to cause brain damage, typical psychological or emotional maltreatment may not be sufficient to impair basic memory function. Studies on the self-referential memory have demonstrated that even in individuals with amnesia or Alzheimer’s disease, the memory advantage of self-referential encoding remains intact, indicating that self-representations exhibit a strong stability in memory and are not easily disrupted (44, 45). In contrast, the mother, as a significant object of self-expansion, may similarly maintain a high degree of stability in her representation. Because individuals who experience psychological maltreatment do not have access to secure and stable emotional support, they must allocate more cognitive resources to monitor potential threats and maintain personal safety, thereby sustaining self- and mother-related representations (46).

Experiment 2 further revealed that the modulatory effect of emotional valence primarily occurred in the control group rather than the psychological maltreatment group, with both SRE and MRE in the control group showing a negativity preference. We speculate that the lower emotional sensitivity observed in the psychological maltreatment group may be related to a functional avoidance emotion regulation strategy. Valentino et al. (47) found that maltreated children exhibited greater overgeneralization in autobiographical memory retrieval compared to the control group, and the level of overgeneralization was significantly positively correlated with negative self-representations. According to the overgeneralization memory model, individuals who experience childhood trauma gradually develop functional avoidance emotion regulation strategies by processing self-relevant emotional details in a more generalized manner, thereby avoiding the impact of specific, negative recollections on their self-concept (48). However, the current results did not reveal a negativity preference in the integration of self and mother representations for individuals with a history of psychological maltreatment. Instead, the correlation analysis results indicated a positivity preference, with individuals who experienced more frequent psychological maltreatment demonstrating stronger memory recall for self-referential and mother-referential information under positive conditions. This collectively suggests that individuals with psychological maltreatment histories may adopt a functional avoidance emotion regulation strategy when processing self- and mother-related negative emotional information. This strategy likely inhibits the effective retrieval of negative situational details related to the self and mother, while enhancing the retrieval of positive situational details to protect the self-concept system from being undermined by negative emotional experiences. In contrast, the control group exhibited a negativity preference. Memory studies have shown that individuals tend to have higher accuracy for R judgments on negative words compared to neutral words, indicating that negative stimuli evoke memories with more contextual details (49). This may attributed to the association of negative stimuli with potential threats, which naturally attract attention and undergo prioritized processing. Consequently, individuals engage in deeper self-referential and mother-referential encoding for negative stimuli, ensuring that contextual details related to the self and mother are effectively retrieved (50).

These results collectively suggest that while experiences of psychological maltreatment during childhood may subjectively reduce the emotional connection between self and mother, they may not impair cognitive differentiation between self- and mother-related information at the perceptual and memory levels, nor do they exhibit a negativity preference at the memory level. However, this study has several limitations, which may explain some of the null findings. First, psychological maltreatment often co-occurs with other forms of abuse, such as sexual and physical abuse, and future studies should control for the effects of these other forms of maltreatment to avoid confounding results. Second, the study had a predominance of female participants, which may limit the generalizability of the findings. For example, female participants with a history of psychological maltreatment may report more negative emotional and behavioral effects (51). Future research should better balance participant gender or investigate the potential gender differences in these effects. Third, the study did not assess the source of the abuse. Given that psychological maltreatment emphasizes the ongoing and harmful interaction patterns between caregivers and children, the source of the abuse is a crucial factor influencing psychological maltreatment (6). The null results observed in this study may be attributed to a lower degree of maternal involvement in the maltreatment, and future studies should consider including this factor. Finally, although the screening criteria for both the psychological maltreatment and control groups in this study were consistent with those used in previous research (2, 34), these criteria still lack normative evidence. This may, to some extent, increase classification biases such as false negatives and false positives, thereby reducing the reliability of the result inferences. Therefore, future studies should be more cautious in selecting screening criteria or establish them based on normative data.

## Data Availability

The original contributions presented in the study are included in the article/**Supplementary Material**. Further inquiries can be directed to the corresponding author.
